# Evolution analysis of EUV radiation from laser-produced tin plasmas based on a radiation hydrodynamics model

**DOI:** 10.1038/srep45212

**Published:** 2017-03-23

**Authors:** M. G. Su, Q. Min, S. Q. Cao, D. X. Sun, P. Hayden, G. O’Sullivan, C. Z. Dong

**Affiliations:** 1 Key Laboratory of Atomic and Molecular Physics & Functional Material of Gansu Province, College of Physics and Electronic Engineering, Northwest Normal University, Lanzhou 730070, China; 2 School of Physical Sciences and National Centre for Plasma Science and Technology, Dublin City University, Glasnevin, Ireland; 3 School of Physics, University College Dublin, Belfield, Dublin 4, Ireland

## Abstract

One of fundamental aims of extreme ultraviolet (EUV) lithography is to maximize brightness or conversion efficiency of laser energy to radiation at specific wavelengths from laser produced plasmas (LPPs) of specific elements for matching to available multilayer optical systems. Tin LPPs have been chosen for operation at a wavelength of 13.5 nm. For an investigation of EUV radiation of laser-produced tin plasmas, it is crucial to study the related atomic processes and their evolution so as to reliably predict the optimum plasma and experimental conditions. Here, we present a simplified radiation hydrodynamic model based on the fluid dynamic equations and the radiative transfer equation to rapidly investigate the evolution of radiation properties and dynamics in laser-produced tin plasmas. The self-absorption features of EUV spectra measured at an angle of 45° to the direction of plasma expansion have been successfully simulated and explained, and the evolution of some parameters, such as the plasma temperature, ion distribution and density, expansion size and velocity, have also been evaluated. Our results should be useful for further understanding of current research on extreme ultraviolet and soft X-ray source development for applications such as lithography, metrology and biological imaging.

Laser-produced plasmas (LPPs) have been an attractive research topic because of their potential use as standard laboratory ion sources^1^,^2^ and pulsed short wavelength light sources^3^,^4^ for important applications such as extreme ultraviolet (EUV) lithography^5^,^6^,^7^, EUV metrology^8^,^9^ and surface treatment and modification^10^,^11^. Especially, in the past ten years, laser-produced tin plasmas have been widely investigated because their compactness and high emissivity around 13.5 nm makes them an attractive extreme ultraviolet light source^12^. Experimental work which includes studies of LPPs starting from simple planar targets to droplet targets, delivered at very high frequencies, has been performed^13^,^14^, while theoretical work has covered topics from the investigation of fundamental atomic transitions contributing to the 13.5 nm band to ion dynamics and energy transport in LPPs^15^. These studies have greatly broadened the understanding of the emission characteristics of LPPs in the EUV region and promoted the further application of LPPs as light sources. Recently, LPP sources emitting soft X-rays in the water window are being developed for use in a number of novel microscope designs for water window imaging and cell tomography^16^,^17^. For these applications, optimization of conversion efficiency and/or brightness are the topics of major concern, while knowledge of ion distributions and velocities are essential in designing effective debris elimination schemes and evaluating the potential effects of ion impact on multilayer mirror surfaces^18^.

In previous studies, tin spectra in the 13.5 nm region, recorded with Nd:YAG lasers, show a complicated spectral profile with a broad re-absorption band and several pronounced dips since the opacity effect is so high for 13.5 nm light that EUV photons emitted from the plasma core are absorbed strongly in the expanding low-temperature plasma periphery^19^. That is to say, the true spectral profiles of tin ions in optically thick plasmas are distorted to yield the observed profile by self-absorption features owing to the opacity effect. Meanwhile, the fact that more than tens of thousands of individual lines contribute to the quasi-continuum band or unresolved transition array (UTA) spectral profile in the EUV region presents a challenge for theoretical calculations of accurate energy levels and plasmas dynamics^20^.

Simulation of the plasmas dynamics plays a crucial role in analyzing and interpreting experimental measurements. Radiation-hydrodynamics models are often used to study the dynamics of laser-produced plasmas and high-current z-pinch plasmas, as well as in the study of laser-driven inertial fusion and short-pulse laser interactions^21^,^22^,^23^,^24^,^25^. In recent years, many research groups have, experimentally and theoretically, carried out research on the dynamical properties of LPPs, which mainly involve the characterization of plasma expansion during the interaction of the laser with both target material and plasma^26^,^27^. However, most of these studies concentrate on the time and space evolution behavior of atoms/ions of low Z elements interacting with a background ambient gas^28^,^29^,^30^,^31^. Only limited research on highly charged ions of middle Z elements has been reported to date. For example, MacFarlane *et al*.^32^ presented a one-dimensional radiation magneto hydrodynamics code with inline atomic kinetics modeling to simulate the dynamic evolution of laser-produced plasmas and z-pinch plasmas. Larsen *et al*.^33^ developed a one-dimensional radiation hydrodynamics code (HYADES) to simulate laboratory experiments on dense plasmas driven by intense sources of energy. White *et al*.^34^ investigated the effect of laser pulse width and spatial profile on conversion efficiency over a range of power densities using a two-dimensional radiative magneto hydrodynamic (MHD) code and analyzed the self-absorption effects in the plasma edge. Nishihara *et al*.^27^ developed an improved power balance model to investigate the optimization of laser and target conditions in order to obtain the high conversion efficiency. Hara *et al*.^26^ used a two-dimensional radiation hydrodynamics simulation (STAR-2D) code^35^ to study the temporal evolution of parameters in a high-brightness EUV source. From the foregoing work it is seen that some comprehensive models have been developed and used to analyze the evolution of plasmas and predict the radiation transport. However, models which can systematically analyze and simulate the EUV spectra involved that correctly reproduced self-absorption spectral features from LPPs of heavy elements, are still scarce.

In our previous work^36^, we reported a simplified radiation hydrodynamic model combined with a local thermodynamical equilibrium (LTE) model and successfully achieved the spatio-temporal evolution analysis of a low Z laser-produced Si plasma. However for more complicated highly-charged ions from middle- and high-Z elements, in most cases there is a limitation for solving the Saha equations because of inaccuracies owing to incomplete atomic data. Colombant and Tonon^37^ presented numerical results based on collisional-radiative equilibrium for the characteristics of highly-charged ions in laser-produced plasmas, which avoids lengthy calculations in solving the rate equation and this model has been widely used to analyze the spectra from laser-produced plasmas.

Therefore, in this work we extend the previous model to investigate more complicated spectral features by combining it with the steady-state collisional-radiative model, and explain the self-absorption features observed experimentally in the EUV spectra of highly-charged tin ions. In addition, we predict the plasma evolution by simulating the spectra related to different experimental conditions produced in a series of experiments and reconstruct the dynamics evolution of a LPP expansion. The goal of our present work is to develop a reliable model to study and optimize the radiation characteristics of highly charged ions in other mid- and high-Z plasmas that will be of use to groups working on both ion and light source development.

## Experiments

**Figure 1:**
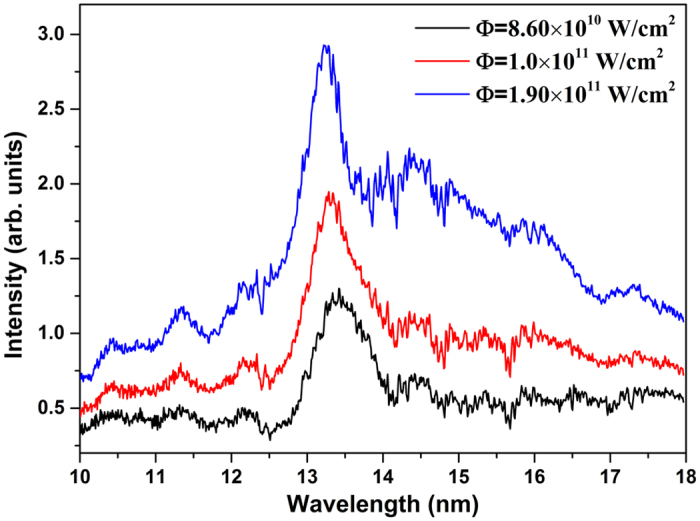
Emission spectra of plasmas formed from solid tin targets, viewed at angles of 45° to the target normal under normal incidence laser irradiation with pulse power densities of 8.60 × 10^10^ W/cm^2^, 1.0 × 10^11^ W/cm^2^, and 1.90 × 10^11^ W/cm^2^.

The EUV emission spectra of highly-charged Sn ions had been used to benchmark the model, which is investigated in the laser power density range of 0.6~1.6 × 10^11^ W/cm^2^. Spectra were recorded at an angle of 45° to the incident laser pulse. Details of the experimental setup and measurements have been reported^20^, and only a brief description is presented here. A Nd: YAG laser with a fundamental wavelength of 1064 nm and pulse width of 15 ns was focused at normal incidence onto solid flat pure tin targets by plano-convex BK7 glass lenses. The range of power densities used was obtained by varying the energy of the incident laser pulse, while maintaining the same focusing conditions. The spectra were recorded on a Jenoptik 0.25 m grazing incidence flat field spectrograph. This spectrograph was fitted with an absolutely calibrated backside illuminated *x*-ray CCD, covering the spectral range 9.5–18 nm. The 36 μm high by 4 mm long piezoelectric entrance slit of the spectrometer was located 1.65 m from the point of plasma formation, with an additional optical path length of 0.48 m between the entrance slit and the CCD detector. This led to a solid angle of collection of 3.8 × 10^−8^ sr, with 285 pixels of the CCD exposed to the incoming radiation. As the laser was operated in single shot mode, with the Q-switch synchronised to produce a single laser pulse 0.5 to 1.5 s after sending the TTL trigger to begin the CCD exposure and open the piezoelectric entrance slit. As the entrance slit required 2.5 ms to open fully, this set-up ensured that a fully time-integrated spectrum from a single plasma only was recorded. In this work, to reduce the calculation load, three typical spectra measured at power densities of 8.60 × 10^10^ W/cm^2^, 1.0 × 10^11^ W/cm^2^, and 1.90 × 10^11^ W/cm^2^ were taken as examples, as shown in Fig. 1.

## Atomic data

Hartree-Fock (HF) approximation calculations with the Cowan codes were used to generate the required atomic data for the spectral analysis and simulation^38^. Configuration interaction (CI) between the 4d^n^, 4d^n−1^4f, 4d^n−1^5p, 4d^n−1^5f, and 4p^5^4d^n+1^ configurations was taken into account since CI effects considerably change the wavelength and intensity of EUV lines. A total of 37,858 lines of the dominant transition arrays 4p-4d, and 4d-4f, 5f, 5p from Sn^6+^ to Sn^13+^ ions have been included in the calculations in order to explain the observed features of EUV spectra.

## Details of the radiation hydrodynamics model

**Figure 2:**
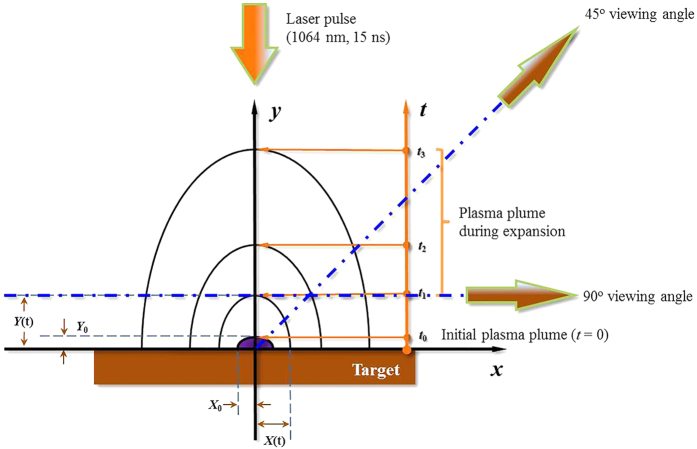
The geometry of the numerical simulations. *X*_0_ and *Y*_0_ present the sizes of initial plasma plume parallel to (*x* axis) and perpendicular to (*y* axis) the target surface, respectively. The values of *X*_0_ and *Y*_0_ are taken as 0.03 cm and 0.04 cm for the power densities of 8.60 × 10^10^ W/cm^2^, 0.035 cm and 0.045 cm for 1.0 × 10^11^ W/cm^2^, and 0.04 cm and 0.05 cm for 1.90 × 10^11^ W/cm^2^. The simulations at 45° and 90° viewing angles to the incident laser pulse are performed for comparison, in which the line integral of the emission at 45° viewing angle is considered from the laser spot to the periphery of the plume, while for 90° the line integral involved from one side to the other of the periphery.

The understanding of the laser-produced plasma expansion is a prerequisite for the evolution analysis of spectral profiles in time and space. The expansion of the plasma plume is closely related to the plasma formation processes. But the formation of the plasma plume is quite a complicated problem, and a complete treatment would involve lengthy calculations and introduce some uncertainties, which would be difficult to evaluate because of the lack of available experimental data. It should be noted, however, that the characteristic time (~microseconds) of the plasma expansion in vacuum for a LPP is longer than the duration of the laser pulse, which permits a separate consideration of the formation and the expansion phases of the plasma plume. In our work, we ignore the formation of the plasma plume and the initial plasma shape is assumed as a semi-ellipsoid, (*x/X*_0_)^2^ + (*y/Y*_0_)^2^ ≤ 1 and *y* ≥ 0, as shown in Fig. 2. Only three numerical parameters are used to describe the initial plasma expansion stage: the initial dimensions of the plume, *X*_0_ and *Y*_0_, the density of the plasma, *n*_*0*_, and the initial temperature *T*_*0*_ of the plasma. Typical values of *X*_0_, *Y*_0_ and *n*_*0*_ can be assessed experimentally for laser-produced plasmas, *T*_*0*_ is determined by the laser pulse energy^39^. The initial distributions of plasma temperature and ion density follow Gaussian distributions^40^,^41^, and the pressure at the plasma boundary is zero.

Here, the radiation hydrodynamics model is established based on the fluid dynamics equations and the radiative transport equation in a Cartesian coordinate system. The fluid dynamics equations are composed of the continuity equation, momentum conservation equation and energy conservation equation. The change of the radiation intensity can be expressed by the radiation transport equation. Their forms are as follows^42^:


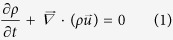



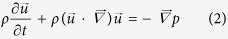



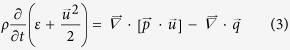



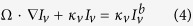


where *ρ* is the plasma density, *u* is the expansion velocity of the plasma, *q* is the total energy loss^42^. *I*_*v*_ and 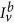 are the plasma radiation intensity and the black body radiation intensity, respectively. *κ*_*v*_ is the effective absorption coefficient that can be expressed as *κ*_*v*_ = *κ*_*ff*_ + *κ*_*fb*_ + *κ*_*bb*_, in which *κ*_*ff*_, *κ*_*fb*_, and *κ*_*bb*_ are the free-free, bound-free, bound-bound effective absorption coefficients^43^.

Assuming that the expansion of plasmas follows the ideal gas law, the local pressure, *p*, and the local internal energy density, *pε*, can be expressed as ref. 42:


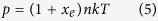



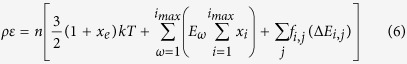


where *kT* is the plasma temperature. *x*_*e*_ is the average ionization degree, *x*_*i*_ is the degree of *i*^*th*^ ionization, *E_ω_* is the minimum energy required for the ionization. Δ*E*_*i,j*_ is the excitation energy with respect to the ground state energy of each *i*^*th*^ ion. Excitation fractions *f*_*i,j*_ are defined by 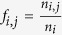. Where *n*_*i,j*_ refers to the *j*^*th*^ excitation state of the *i*^*th*^ ionization stage ion.

The ion charge state distributions were calculated from the steady-state collisional-radiative model, as described by Colombant and Tonon^37^ referred to earlier. Here, a set of rate equations are solved by determining the fractional population of each ion species as a function of electron temperature and electron density. These equations satisfy the steady-state condition for *n*_*z*_, the number density of charge state *Z*, given by


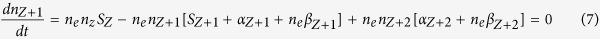


where the quantities *S, α* and *β* are the collisional ionization coefficient, the radiative-recombination coefficient and the three-body recombination coefficient^37^. These coefficients depend on electron temperature, electron density, the ionization potential and number of equivalent outer shell electrons for each charge state. The solution for physical quantities such as electron density, ion density and average ionization degree can be obtained by combining the conservation equation of ion number, 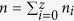, and the charge conservation equation, 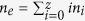.

## Description of experimental spectral characteristics

**Figure 3:**
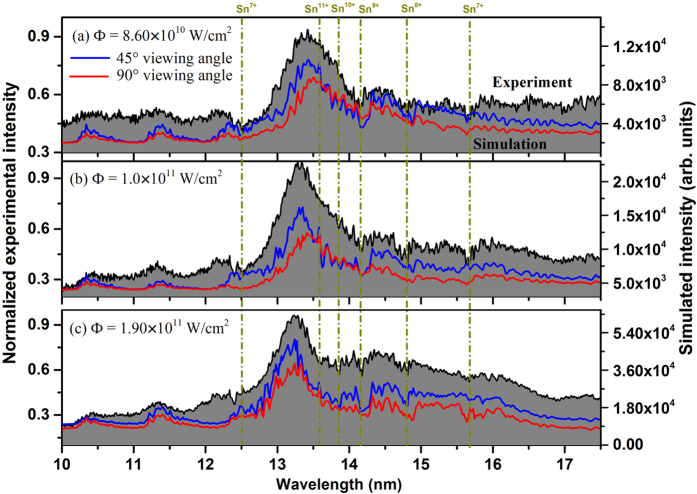
Comparisons of spectral profiles between the experimental and two simulated results based on the radiation hydrodynamics model, in which the black line represents the experimental spectra detected at a 45° viewing angle, and the red and blue lines present the simulated results for 90° and 45° viewing angles, respectively. (**a**), (**b**) and (**c**) represent cases at laser power densities of 8.60 × 10^10^ W/cm^2^, 1.0 × 10^11^ W/cm^2^, and 1.90 × 10^11^ W/cm^2^. Six vertical dotted dash lines point out the absorption dips in the EUV spectra.

The black lines in Fig. 3 (denoted by the black solid line) show the spectra of pure tin at three power densities of 8.60 × 10^10^ W/cm^2^, 1.0 × 10^11^ W/cm^2^, and 1.90 × 10^11^ W/cm^2^, which were recorded at a 45° viewing angle. It is clearly seen that the spectral profile consists of a broad quasi-continuum band and six absorption dips near 13.61 nm, 13.85 nm, 14.17 nm, 14.76 nm, 15.65 nm and 16.90 nm overlapped on a background continuum, and with increasing power density the peak of the broad quasi-continuum band initially centered near 13.50 nm shifts to 13.25 nm and its width become narrower. Although the complexity of the spectra, which involve ions with an open 4d subshell, could potentially make interpretation of this spectral behavior difficult, fortunately, previous experimental and theoretical work provides information on the line distributions in tin ion spectra from the sixth to thirteenth ionization stages^20^. These experimental spectra should be helpful for the identification of the spectral structure in the EUV spectral regime and for theoretical simulation.

## Comparison between experimental and theoretical results

As the experimental spectra were the time-integrated, the signal reaching the detector is the sum of signals from plasma volumes with different temperatures and densities due to the highly inhomogeneous and transient nature of LPPs. For comparison with the experimental spectra, the simulated time-resolved spectra (gate width of 5 ns in 5 ns steps) are summed over the duration of the emission to replicate the time-integrated measurements. Figure 3 (a) and (c) show the comparisons of normalized spectral profiles between the experimental spectra and two simulated results based on the radiation hydrodynamics model. Here we show predicted spectra (denoted by red lines) expected for a 90° viewing angle near the target surface for comparison with the spectra (denoted by blue lines) for a viewing angle of 45°. It is clearly seen that there is good agreement between the experimental and simulated profiles for the 45° viewing angle, especially in relation to the agreement of the positions and profiles of the main emission peaks and also for the positions and profiles of several absorption dips. The positions of these dips have also been labeled in this figure with the vertical dotted dash lines. In addition, it is obvious that there are some differences between the simulated results for 45° and 90° because of the different optical absorption paths considered in the calculations. It is found that the self-absorption, at least for the ion stages responsible here, at 45° is larger than at 90°. This shows that the gradients of temperature and density encountered at 45° are larger than those at 90° and also that the optical absorption length is greater for the ion stages responsible.

### Line distribution profiles and absorption profiles for 4p-4d, 4d-4f and 4d-5f transitions

**Figure 4:**
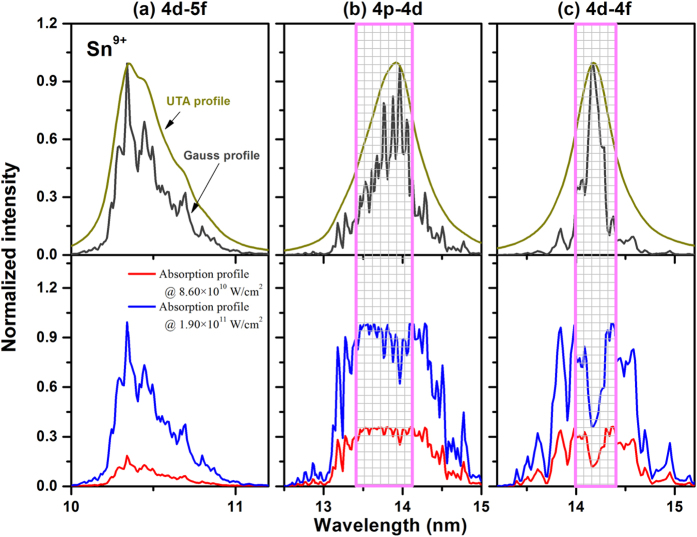
Theoretical simulation and comparison of laser-produced Sn spectra. The dark yellow and dark gray solid lines in the upper panel present the UTA profile and Gauss profile of calculated lines, respectively. While the blue and red lines in the lower panel present the simulated absorption profiles at the pulse power densities of 8.60 × 10^10^ and 1.90 × 10^11^ W/cm^2^, respectively.

In order to show more clearly the origin of self-absorption bands and dips, we take the Sn^9+^ ion as an example. Figure 4 shows the normalized spectral profiles of 4d-5f, 4p-4d and 4d-4f transition arrays obtained by using the UTA formalism and Gaussian broadening method, and the corresponding normalized calculated profiles obtained at laser power densities of 8.60 × 10^10^ and 1.90 × 10^11^ W/cm^2^, respectively. It is obvious that the 4d-5f profile in Fig. 4(a) contains a large contribution from the strongest emission feature, but the 4p-4d profile in Fig. 4(b) and 4d-4f profile in Fig. 4(c) show an obvious coexistence between self-absorption and emission features. The important absorbing regions are noted with a rectangular grid filled box for clarity. It is obvious that the absorption profiles are the opposite of their emission Gaussian profiles. From the point of view of spectral intensity, the spectral intensities for 4p-4d and 4d-4f transition arrays allowing for absorption are almost two-thirds those of the integrated intensity in the absence of absorption. Thus the total spectral emission becomes weaker as a consequence of absorption. The spectral feature outside the box in Fig. 4(b) and (c) presents the emission from some weaker transitions, which are close in intensity to the spectral background. So self-absorption reduces the contrast between absorption and emission features and in effect smoothen the spectrum. For the 4p-4d and 4d-4f transitions the wings of the original spectral profiles are effectively raised relative to the intensity at the centres. To some extent, the full width at half maximum (FWHM) of the normalized total profile for absorbed spectra is larger than that of the normalized emission profile in the absence of self-absorption effects. This “opacity broadening” is also an important factor in preventing accurate spectral identification. In addition, there is an obvious discrepancy at 14.17 nm between experimental and simulated spectra. It shows the calculated spectral width to be broader and deeper than the experimental width. This disagreement may arise from the assumption of a simple excited state population given by a normalized Boltzmann distribution among the excited states. A further detailed consideration of the excited energy level population calculation is now in progress.

## Contour images of plasma temperature in the plasma expansion

**Figure 5:**
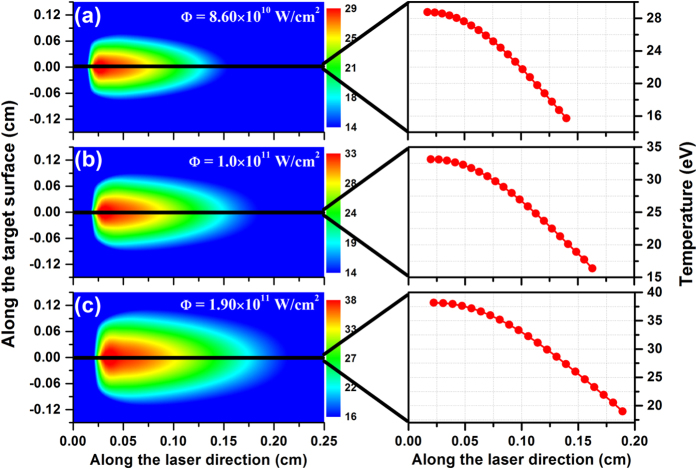
Contour images of plasma temperature in laser-produced tin plasmas calculated at the time delay of 15 ns and at the pulse power densities of 8.60 × 10^10^ W/cm^2^ (**a**), 1.0 × 10^11^ W/cm^2^ (**b**), and 1.90 × 10^11^ W/cm^2^ (**c**), respectively. The graphs on the right side show the varieties of plasma temperature along the laser direction.

**Figure 6:**
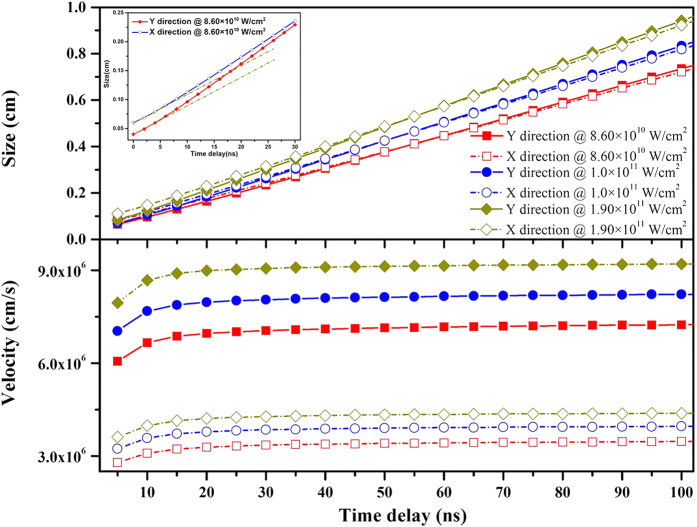
The evolution of the plasma edge velocity and radius perpendicular to (y axis) and parallel (x axis) to the target surface, respectively. The dark yellow dashed line in the inset shows the non-linearity at the early stage of plasma expansion.

The plasma temperature is a very important parameter in the characterization of any plasma, since it determines the ion charge and energy distributions. To provide an intuitive description for the plasma expansion process, the contour images of plasma temperature at the time delay of 15 ns (corresponding to the calculated peak emission) were obtained, and are shown in Fig. 5. It is clearly seen that with increasing power density the distribution in space of the plasma temperature shows a gradual increase at each position considered. For clarity, the graphs on the right hand side in Fig. 5 show the decrease in plasma temperature along the optical axis at the three power densities mentioned above. Obviously, with increasing power density, the electron temperature at the plasma core changes from 28.8 eV at 0.17 mm in Fig. 5(a) to 38.2 eV at 0.22 mm in Fig. 5(c), while the minimum temperature value at the plasma periphery varies from 15.7 eV at 1.40 mm in Fig. 5(a) to 18.9 eV at 1.89 mm in Fig. 5(c). The plasma core and periphery for the three power densities considered here move gradually further away from the target which shows that the thermal energy of the plasma is rapidly converted into kinetic energy so that the plasma can attain extremely high expansion velocities, which usually lies in a velocity range of 10^6^~10^7^ cm/s. This velocity is in good agreement with the result from Chen *et al*. obtained by using a self-similarity theory^44^, and is also further confirmed by the predicted expansion of the plasma from our model, as indicated in Fig. 6.

## Time evolution of plasma size and velocity of plasma edge

Based on the above experimental and theoretical simulation results, in order to clearly reveal plasma expansion and cooling processes, the predicted time evolution of the plasma size and the velocity of the plasma edge along the target surface (X direction) and opposite to the laser incident direction (Y direction) are given in Fig. 6(a) and (b), respectively. In addition, for clarity, an inset showing the temporal evolution of plasma size at the power density of 8.60 × 10^10^ W/cm^2^ in the time range of 5–30 ns is inserted in order to show non-linearity at the early stage of plasma expansion, in which two dark yellow dashed lines are used to intuitively display the degree of non-linearity.

As can be seen, the plasma sizes in the X and Y directions at the same power density have nearly the same growth trend, and with increasing time, the plasma size along the laser direction is slightly larger than that parallel to the target surface. With increasing power density the size of the plasma obviously increases, and with increasing time, this increase becomes more pronounced. Regarding the velocity of the plasma edge, at the initial stage of plasma expansion the velocity of the plasma edge in the Y direction is about twice that in the X direction. As time increases, both perpendicular and parallel velocities gradually increase for around 20 ns, and then approach constant values. Eventually, the velocity of the plasma edge in the Y direction is about 2.7 times larger than that in the X direction. Meanwhile, it can be seen that the larger the power density, the faster the expansion velocity of the plasma edge. With further expansion of the plasma volume, the plasma temperature drops rapidly and the number of collisions between particles is also reduced. As a consequence, the acceleration of the plasma edge will be reduced rapidly.

## Ion distribution and density evolution of different charge states in the plasma expansion

**Figure 7:**
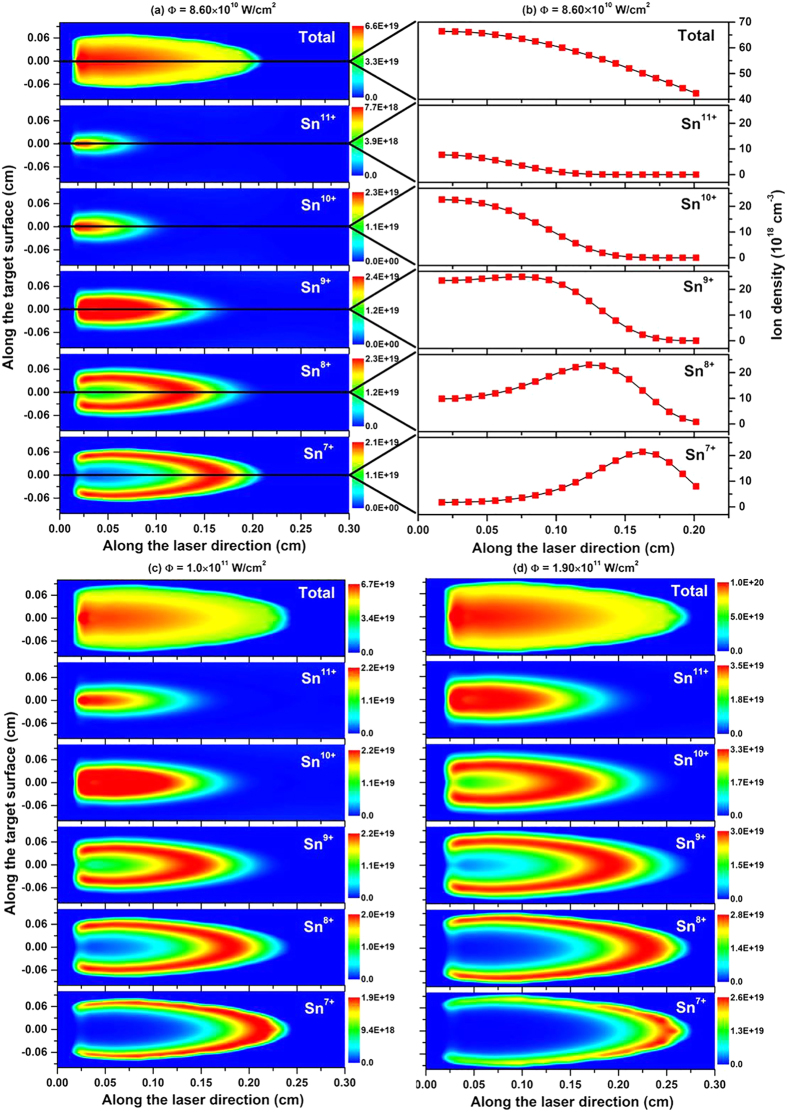
Contour images of total ion number density and ion number density at the time delay of 15 ns for each individual species from Sn^7+^ to Sn^11+^ at three power densities mentioned above. (**a**) and (**b**) show the calculated distributions at the power density of 8.60 × 10^10^ W/cm^2^ and the corresponding density evolution of different ions along the laser direction. (**c**) and (**d**) show the calculated distributions for the power densities of 1.0 × 10^11^ W/cm^2^ and 1.90 × 10^11^ W/cm^2^, respectively.

In order to gain insight into the ion distribution and density evolution of different charge states in the process of plasma expansion, the contour images of total ion number density and ion number density at the time delay of 15 ns for each individual species from Sn^7+^ to Sn^11+^ for the three power densities chosen are indicated in Fig. 7. Figure 7(a) and (b) show the calculated distributions for a power density of 8.60 × 10^10^ W/cm^2^ and the evolution of different ion populations along the Y axis, respectively, for clarity. From this figure, we can clearly determine the ion distribution of different charge states at a specific moment. The higher the degree of ionization, the closer it is to the target surface and the core of plasma. At distances further from the target, the degree of ionization rapidly decreases because of recombination, and the lowest ion stages are moved from the core to the edge of plasma. From this figure, one can easily find the dominant ion distribution at a certain space position. In addition, it is clearly seen that the contour image of total ion distribution should be overlapping layer by layer from these individual ion distributions. It can be seen that with increasing power density, (Fig. 7 (c) and (d)) the total ion density increased from 6.7 × 10^19^ to 1.0 × 10^20^ cm^−3^, the dominant ion moved from Sn^9+^ to Sn^11+^. and the maximum distance at which ions are located along the Y axis increased from 2.11 mm to 2.61 mm. This spatial distribution also further explains the time evolution of Fig. 6. From the plasma core to the edge, there is a clear plasma temperature and density gradient, it is also confirmed that the plasma is highly inhomogeneous and transient.

**Figure 8:**
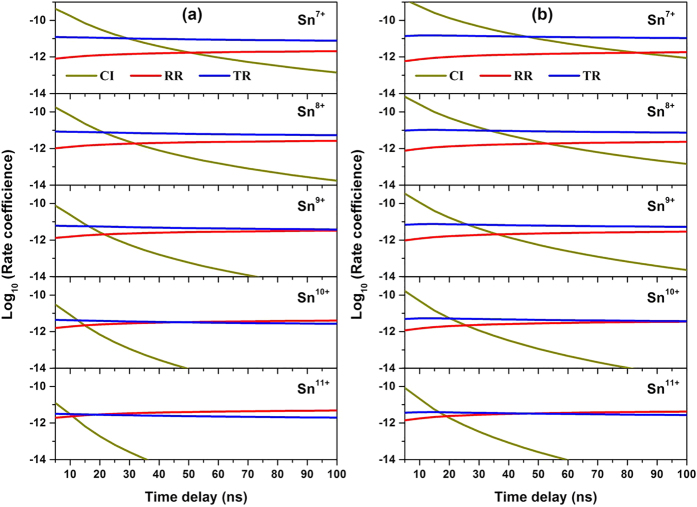
The variation of three dominant rate coefficients of Sn7^+^ to Sn11^+^ ions as a function of time delay at 8.60 × 10^10^ W/cm^2^ and 1.90 × 10^11^ W/cm^2^, respectively. CI, RR and TR refer to the collisional ionization, radiative recombination and three-body recombination coefficients.

Figure 8 (a) and (b) shows the variation of three dominant rate coefficients of Sn^7+^ to Sn^11+^ ions as a function of time delay at 8.60 × 10^10^ W/cm^2^ and 1.90 × 10^11^ W/cm^2^, respectively. The arrangement of ionization stages from top to bottom in these figures is from Sn^7+^ to Sn^11+^. It is obviously seen that with increasing time delay the collisional ionization (CI) coefficient rapidly decreases while the radiative recombination (RR) and three-body recombination (TR) coefficients monotonically increase, and the higher the degree of ionization, the weaker the contribution of the collisional ionization process to ion population. With increasing ionization, the contribution of the radiative recombination process gradually exceeds that of three-body recombination process. Comparing Fig. 8(a) and (b), we also find that the influence of laser power density on the ionization process is greater than on recombination processes, which increases the degree of ionization and maintains the ionization process for a longer time. Thus, at the initial stage of plasma expansion the ionization process dominates, while at the cooling stage the recombination processes are dominant.

## Conclusion

In conclusion, a simplified radiation hydrodynamics model was developed to explain the complicated self-absorption profiles in EUV spectra of highly-charged tin ions. The origin of self-absorption bands and dips around 13.5 nm in Sn spectra have been successfully simulated and explained. The evolution images of laser produced Sn plasmas at different power densities have been reconstructed. These investigations can give a more intuitive physical picture of the distributions of temperature and ion/electron density within the expanding plume. The results are helpful for a more detailed understanding of the spectral features and hydrodynamics evolution for highly charged ions of the mid- and high-Z elements. More importantly, it will be of use to groups working on ion and light source development.

## Additional Information

**How to cite this article:** Su, M. G. *et al*. Evolution analysis of EUV radiation from laser-produced tin plasmas based on a radiation hydrodynamics model. *Sci. Rep.* **7**, 45212; doi: 10.1038/srep45212 (2017).

**Publisher's note:** Springer Nature remains neutral with regard to jurisdictional claims in published maps and institutional affiliations.
